# Our Medical College

**Published:** 1867-12

**Authors:** 


Our Medical College.—The coming session, 1867–8, of our Medical Institution, offers advantages to the student equal to any institution of the country. Our *corpse* of Professors are men of experience as Teachers and Practitioners, and western in their energies and ideas. Our appliances are, in all the departments, every way equal to the wants of the profession and student. We labor to qualify young men for the full discharge of all professional duties—not theoretically alone, but practically. We teach not only from lectures, but by daily examinations and illustrations—leaving practical facts indelably impressed upon the mind of the student. The cost, considering the length of the session and number of lectures, is less than at any other of our regular Colleges. Our aim has always been, to save time and money for the student, and give him all the advantages of a thorough medical edncation. This we claim for the *Medical Department of the Iowa State University.*

